# Integrated microtia and aural atresia management

**DOI:** 10.3389/fsurg.2022.944223

**Published:** 2022-12-26

**Authors:** Mai Thy Truong, Yi-Chun Carol Liu, Jocelyn Kohn, Sivakumar Chinnadurai, David A. Zopf, Melissa Tribble, Paul B. Tanner, Kathleen Sie, Kay W. Chang

**Affiliations:** ^1^ Department of Otolaryngology-Head and Neck Surgery, Stanford University, Palo Alto, CA; ^2^ Department of Otolaryngology-Head and Neck Surgery, Baylor College of Medicine, Houston, TX; ^3^ Division of Pediatric Otolaryngology, Texas Children's Hospital, Houston, TX; ^4^ Department of Otolaryngology-Head and Neck Surgery, Stanford Children's Hospital/Packard Children's Health Alliance, Walnut Creek, CA; ^5^ Children's Minnesota Pediatric Otolaryngology and Facial Plastic Surgery, Minneapolis, MN; ^6^ Department of Otolaryngology-Head and Neck Surgery, University of Minnesota, Minneapolis, MN; ^7^ Department of Otolaryngology-Head and Neck Surgery, University of Michigan, Ann Arbor, MI, USA; ^8^ Department of Audiology, Lucile Packard Children's Hospital at Stanford University, Palo Alto, CA; ^9^ Department of Surgery, University of Utah, Facial Prosthetics, Salt Lake City, UT, USA; ^10^ Department of Otolaryngology-Head and Neck Surgery, University of Washington/Seattle Children's Hospital, Seattle, WA

**Keywords:** microtia, aural atresia, aural atresia surgery, microtia surgery, auricular reconstruction, ear canal stenosis, bone conduction implant (BCI), ear canal reconstruction

## Abstract

**Objectives:**

To present recommendations for the coordinated evaluation and management of the hearing and reconstructive needs of patients with microtia and aural atresia.

**Methods:**

A national working group of 9 experts on microtia and atresia evaluated a working document on the evaluation and treatment of patients. Treatment options for auricular reconstruction and hearing habilitation were reviewed and integrated into a coordinated care timeline.

**Results:**

Recommendations were created for children with microtia and atresia, including diagnostic considerations, surgical and non-surgical options for hearing management and auricular reconstruction, and the treatment timeline for each option. These recommendations are based on the collective opinion of the group and are intended for otolaryngologists, audiologists, plastic surgeons, anaplastologists, and any provider caring for a patient with microtia and ear canal atresia. Close communication between atresia/hearing reconstruction surgeon and microtia repair surgeon is strongly recommended.

## Objectives

• Standardize the evaluation, including hearing evaluation, for a newborn with microtia and aural atresia
• Review hearing amplification options, including worn devices and surgically implanted devices for patients with microtia and aural atresia
• Review reconstructive options for microtia and the timeline that each option entails
• Review issues concerning aural atresia repair, especially in coordination with microtia repair.
• Create a coordinated timeline for hearing management and microtia reconstruction to reduce the total number of surgeries required and avoid potential deleterious impacts of hearing surgeries on microtia repair.

## Target patient population

Pediatric and adult patients with microtia and aural atresia

## Intended Users

(a) Otolaryngologists: pediatric otolaryngologists, otologists, facial plastic surgeons
(b) Plastic surgeons
(c) Oral/Maxillofacial surgeon
(d) Audiologists
(e) Anaplastologists
(f) Pediatricians
(g) Any provider caring for patients with microtia, aural atresia, and craniofacial abnormalities

## Methods

Each of the authors provided expert opinions. The primary authors (MT, KC) wrote a draft of the manuscript with additional input provided by the contributing authors in specific niche interests and practice areas. A final consensus was obtained on the management recommendations reviewed and approved by all contributors.

## Introduction

### What is microtia?

**Figure 1:**
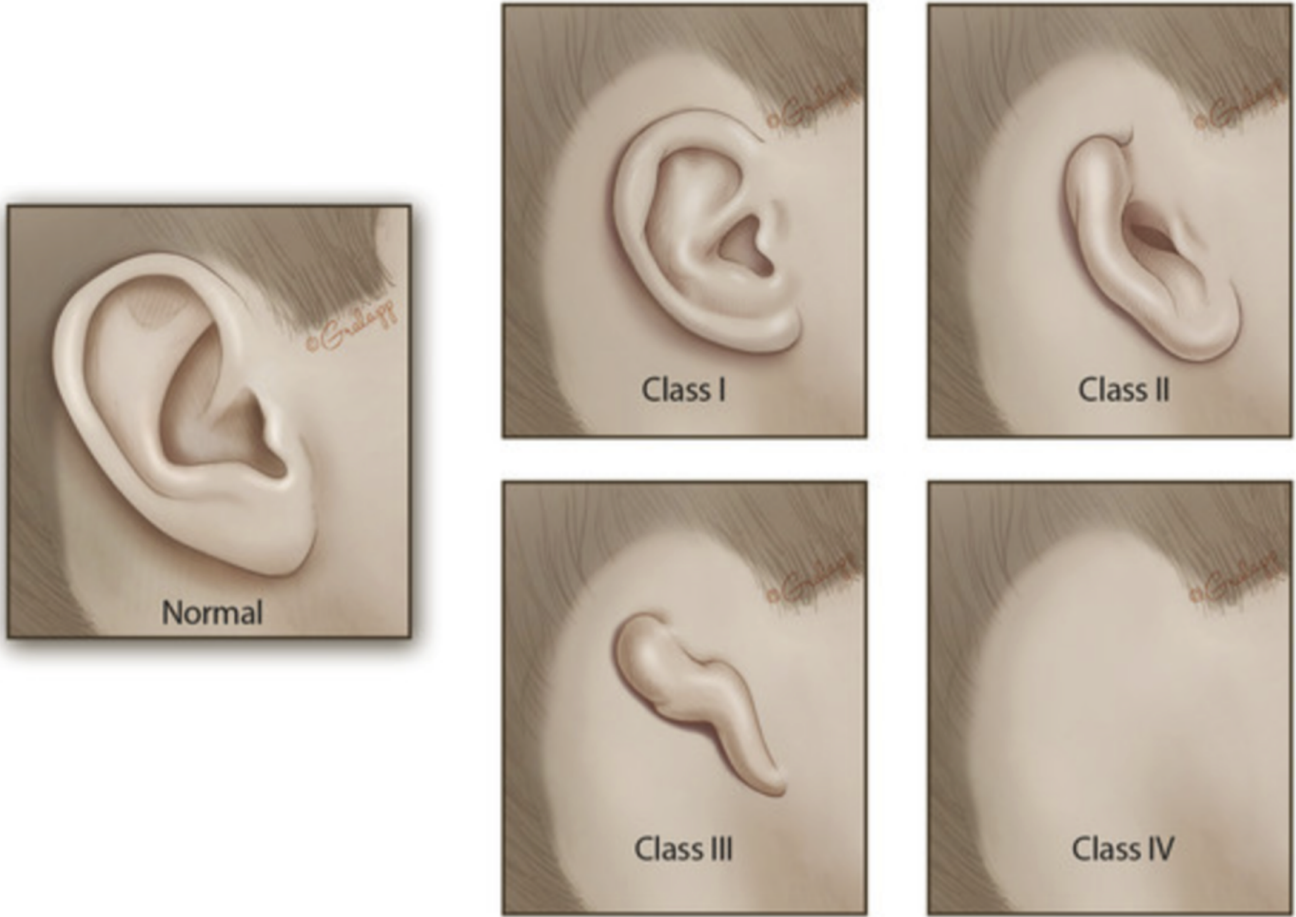
Classification of Microtia Severity.

Microtia is a congenital malformation of the outer ear or pinna whereby the ear is reduced in size, has underdeveloped landmarks, or is misshapen (1). Microtia is often associated with the absence of the external auditory canal (canal atresia or aural atresia) or an extremely narrow ear canal (canal stenosis). Classically, microtia is classified into four grades, ranging from mild malformation to the complete absence of the external ear (Figure 1). The most common presentation is grade 3, consisting of significant hypoplasia of the entire cartilaginous framework with a lobule remnant. Microtia ears can also be described as “lobular-type,” when there is a predominant lobule remnant or “conchal-type,” where a conchal bowl remnant is present. “Conchal-type” microtia is often associated with the presence of a stenotic canal.

Microtia occurs in about 1 in 10,000 births, though rates vary depending on ethnic background (1). Ninety percent of cases are unilateral, with the right side being affected more frequently than the left, and it is more prevalent in males than females. The exact cause of microtia and atresia is unknown, and there may be a variety of different etiologies. There is evidence for both environmental and genetic causes of microtia. This malformation is associated with a syndrome in 35%–55% of patients, many of which have an identified genetic mutation (2). In non-syndromic or sporadic cases, no single genetic mutation has been identified. Maternal exposure to medications during pregnancy, such as retinoids and thalidomide, have been identified (3, 4). Microtia also has an increased incidence in countries at a higher elevation (5). An association between maternal diabetes and microtia has also been suggested (6).

### What is aural atresia?

**Figure 2:**
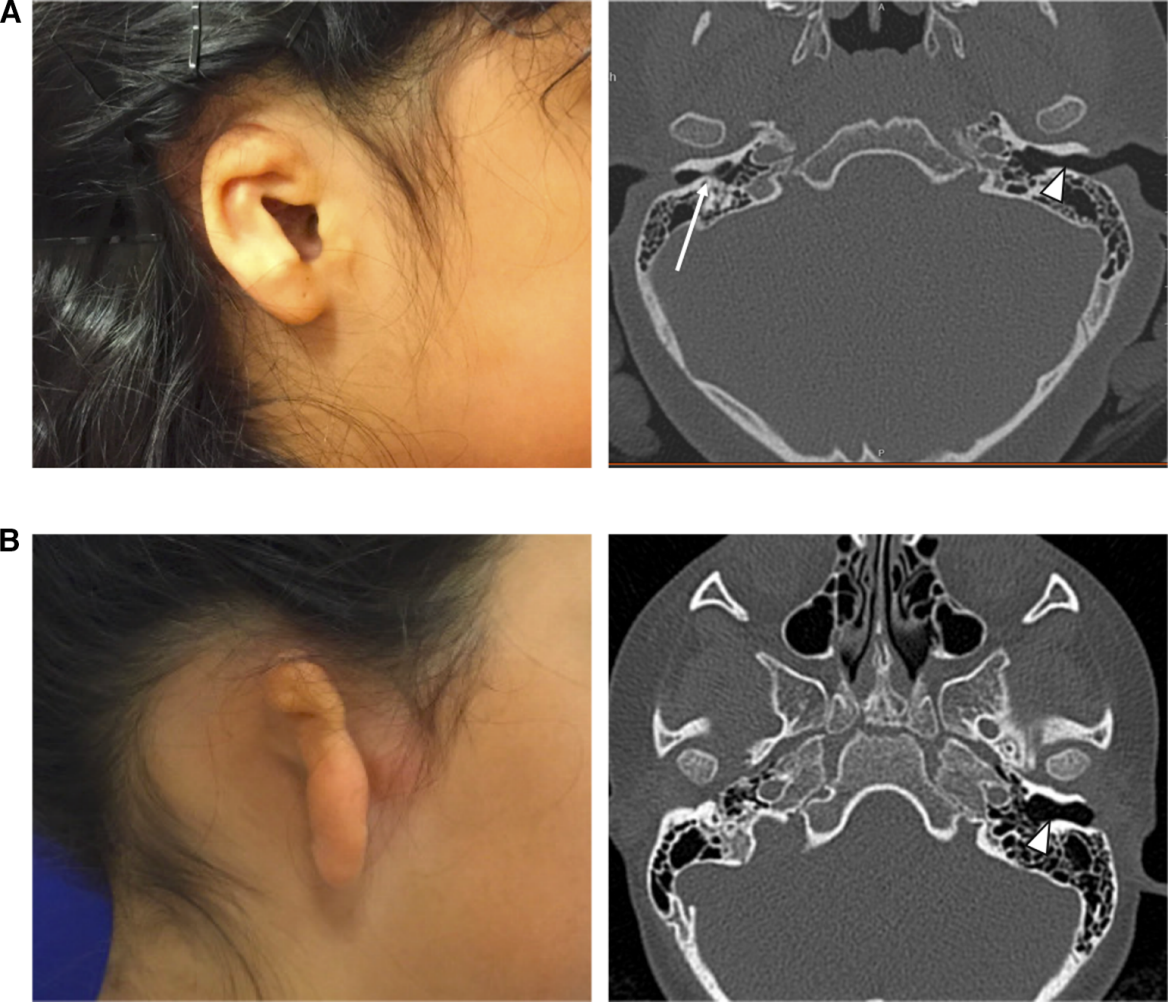
(**A**) Child with visible canal opening on the right, but severe bony external canal stenosis demonstrated on non-contrast CT scan (long white arrow). Normal canal size is noted on the contralateral side (short white arrow). (**B**) Child with complete atresia on the right, demonstrated on non-contrast CT scan. Normal canal size is noted on the contralateral side (short white arrow).

Deficiencies of the ear canal, also called aural atresia, are often present with microtia. Varying degrees of aural atresia can be classified from Type A (Stenosis), in which both the fibrocartilaginous and bony parts of EAC are present but narrow, to Type B (Partial Atresia), in which only some part of the fibrocartilaginous or bony EAC is present with the tympanic membrane being missing or rudimentary, to Type C (Total Atresia), in which both fibrocartilaginous and bony parts of the EAC as well as the tympanic membrane are absent (7). In most cases it is possible to classify the degree of atresia from careful physical exam, however in some unusual cases, the opening to the remnant ear canal may appear only as a tiny pit on the skin, or be hidden by overlying microtic skin remnants. In these cases, a CT scan (Figure 2) can better delineate that a stenotic, epithelium-lined bony ear canal is still present, posing a risk for an eventual canal cholesteatoma.

## Management recommendations

### The workup of a newborn or child with microtia/atresia

History: Clinicians should perform a complete history including ear infections, speech and language milestones, and family history of hearing loss and ear anomalies.

Physical exam: A complete physical exam of the infant should be performed looking for associated syndromic findings (Table 1). It is also essential to monitor the middle ear status in the typically formed ear to ensure optimal hearing is maintained for the better hearing ear.

**Table 1:** Syndromes commonly associated with microtia.

Syndromes associated with Microtia
Oculo-Auriculo-Vertebral Spectrum
-Including Goldenhar syndrome and Hemifacial Microsomia
Town Brocks Syndrome
Branchial-oto-renal Syndrome
Treacher Collins Syndrome
DiGeorge Syndrome (22 q deletion)
Distal 18 q Deletion Syndrome
Nager Syndrome
Fibroblast growth factor 3 deficiency
Lacrimal-auriculo-dento-digital (LADD) Syndrome
CHARGE syndrome
Walker Warburg Syndrome
Klippel-Feil Syndrome
Meier-Gorlin Syndrome

Hearing evaluation: When infants present with microtia and aural atresia, the involved ear(s) will typically fail the newborn hearing screen (NHS). Regardless of the NHS results, these infants should be referred directly to an audiologist to undergo an outpatient diagnostic Auditory Brainstem Response (ABR) test (8). Newborn hearing screening is mandated and performed universally in the United States, but often by non-medical volunteers. Regardless of the presence or absence of an ear canal, the infant with microtia or with a severe auricular malformation should be referred to a pediatric audiologist (or a general audiologist comfortable doing office sleep ABRs) for diagnostic hearing testing.

A diagnostic ABR should be performed as early as possible. The Early Hearing Detection and Intervention (EDHI) program goals recommend a diagnostic ABR by 3 months of age. For clinics and centers meeting this metric, they should strive for a diagnostic ABR by 2 months of age (8). At a minimum, this diagnostic ABR should include thresholds for broadband stimulus such as a click or CE-Chirp, as well as tone burst thresholds between 500 Hz and 4,000 Hz. Air conduction and bone conduction testing should performed. Specifically, masked bone conduction testing will best isolate the response from the cochlea of the affected ear to ensure the bone conduction response is indeed coming from the test ear. In cases of bilateral atresia, only unmasked bone conduction can be measured due to the inability to appropriately mask the bone conduction response. Otherwise, in cases of unilateral ear canal stenosis or atresia, masked bone conduction testing should be performed for the affected ear. Additionally, tympanometry should be performed (1000 Hz tympanometry to demonstrate the mobility of the eardrum and 226 Hz tympanometry to confirm ear canal volume) and distortion product otoacoustic emissions in the ear with a patent ear canal.

**Figure 3:**
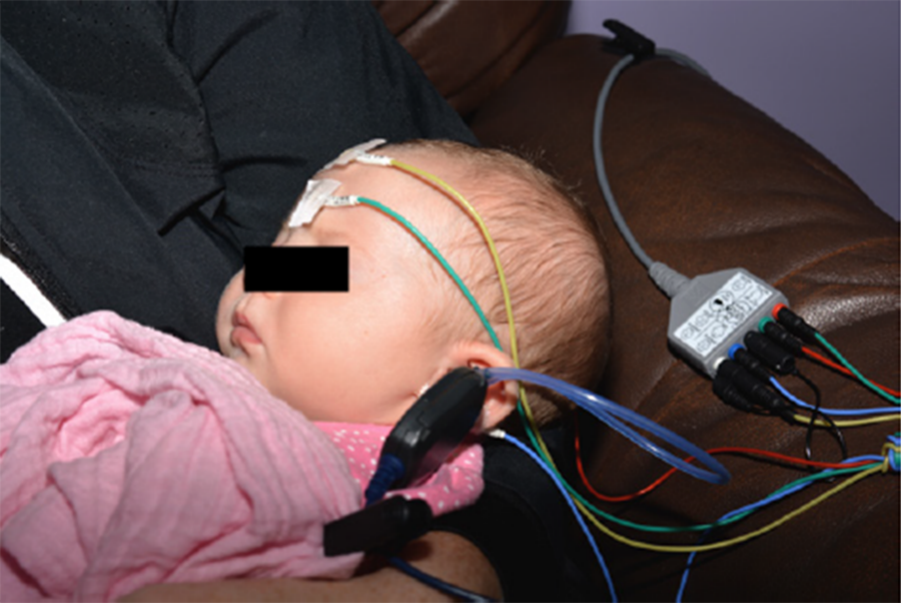
Infant undergoing unsedated (natural sleep) auditory brain stem testing (ABR).

In most infants from birth to up to 6 months of age, this diagnostic ABR can be done while the infant is sleeping without sedation (Figure 3). Beyond six months of age, audiologic assessments should be performed using developmentally appropriate behavioral test methods (visual reinforcement audiometry, conditioned play audiometry, standard audiometry). The behavioral audiologic evaluation is essential to confirm hearing thresholds (by masked bone conduction) for the ear with microtia/atresia as well as confirm and monitor hearing sensitivity for the non-microtic/atretic ear. Children who are challenging to assess behaviorally may require a sedated ABR. Hearing sensitivity in this population should be monitored annually or bi-annually until age 5.

In cases of bilateral microtia and atresia, patients should be referred to audiology as early as possible. Diagnostic audiological assessment should be completed by 2–3 months of age and the infant provided with hearing amplification by 4 months of age and enrolled in early intervention (EI) by 3–6 months of age to optimize speech and language development.

**Figure 4:**
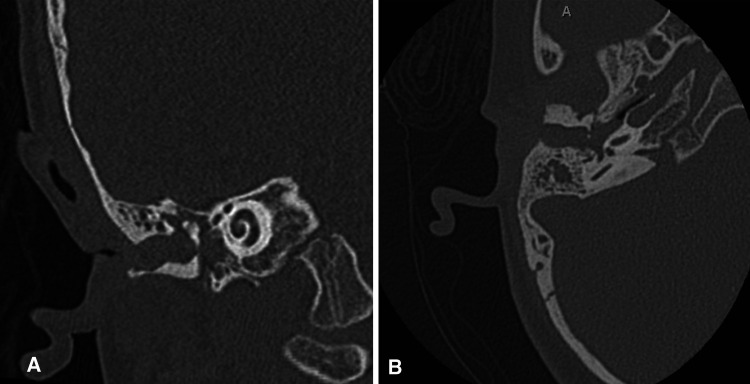
(**A**) Coronal and (**B**) axial non-contrast temporal bone CT scan demonstrating severe EAC stenosis with cholesteatoma.

Imaging studies: A CT scan of the temporal bone is not recommended in the newborn period. CT should be reserved for just before hearing reconstructive surgical options are discussed (approximately age five years, depending on the surgical plan) or if there is a concern for cholesteatoma. A high-resolution CT scan of the temporal bone without contrast is recommended before any microtia reconstruction to assess candidacy for atresia repair and to rule out the presence of canal or congenital middle ear cholesteatoma (Figure 4), though it may not be necessary if the family has absolutely no interest in atresia repair. Clinical concern for cholesteatoma, often seen in cases with severe canal stenosis, may warrant diffusion-weighted imaging (DWI) MRI to help confirm the diagnosis. Renal ultrasound in the newborn period can be performed to rule out kidney abnormalities associated with syndromic causes of microtia (9).

Consultations: Newborns should be referred to audiology and otolaryngology to consult on the child's hearing status. In addition, a genetics referral is recommended if the child appears to be syndromic at birth (Tables 1, 2). These referrals can be made as an outpatient, though many parents are comforted to meet with an otolaryngologist after birth, particularly to address hearing concerns. An adolescent with hemifacial microsomia may require a plastic surgery or oral and maxillofacial surgery (OMFS) referral regarding mandibular hypoplasia and malocclusion issues. This referral may be through a Craniofacial clinic. Parents or patients desiring more information about the actual microtia reconstruction should be referred to an experienced microtia surgeon; this may be a plastic surgeon/craniofacial surgeon, pediatric otolaryngologist, or facial plastic surgeon. Recommend evaluations for speech and language delay and Early Intervention programs that are available from birth to age three years in every state for children with developmental delays and disabilities (10), including isolated hearing loss. After age 3, it is important to pursue an Individualized Education Plan (IEP) or 504 (c) plan through the school system to set the child up for success in the classroom.

**Table 2:** Consultations to consider for newborn/child with microtia and atresia.

Consultations to consider for newborn/child with microtia and atresia
Otolaryngology
(Pediatric) Audiology
Genetics
Craniofacial Clinic
Plastic Surgery
Oral-maxillofacial surgery
Microtia Specialist
Speech pathology

### Counseling for parents of children with microtia

Parents of a newborn with microtia may experience intense emotions, especially guilt and frustration. It is important to counsel parents on theoretical causes of microtia, the hearing impact and then guide them on hearing and reconstructive options and timelines. In most cases, the cause of microtia and atresia is not known. Parental self-blame should be mitigated to the extent possible. Advising parents on ways to talk to the affected child, friends, acquaintances, or other family members about the patient's “little ear” is also paramount to prevent feelings of shame. Families should be encouraged to explore support groups or online communities and forums (e.g., www.earcommunity.org) to minimize feelings of isolation and provide positive examples of how children with microtia and atresia can grow to be productive, healthy adults.

### Hearing device options and recommendations for use in an infant or child with microtia/atresia

Children with microtia/atresia are most commonly found to have conductive hearing loss (CHL) on the side of the microtic ear. The hearing loss is confirmed at the time of the diagnostic audiologic evaluation. Amplification options for children with microtia and atresia can be non-surgical or surgical. Before the age of five years, the non-surgical amplification option is recommended as some surgically implanted device options are approved by the FDA for children of age five years and over.

### Non-surgical hearing amplification options

**Figure 5:**
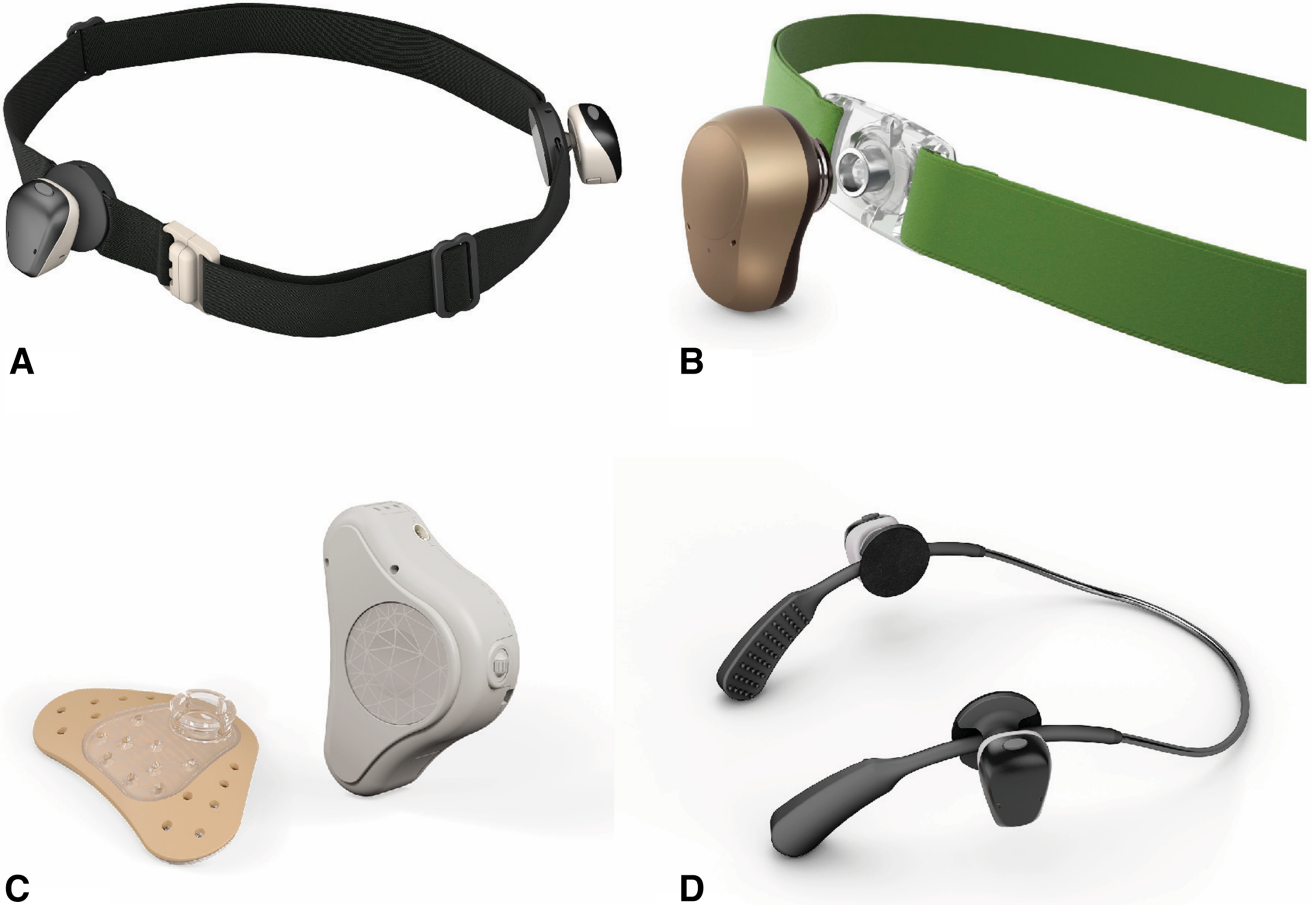
Non-Surgical Bone Conduction Devices. Bone conduction sound processors. (**A**) Cochlear BAHA^®^ with softband. (**B**) Ponto^®^ with softband. (**C**) Med-El ADHEAR^®^ with adhesive gel pad. (**D**) Cochlear BAHA SoundArc^®^.

A variety of worn hearing devices are available to help children with the conductive hearing loss associated with microtia and atresia (Figure 5). Bone conduction sound processors (BCSP) such as Cochlear BAHA® or Ponto can be worn on an accessory known as a softband (11). Another option is the MedEl ADHEAR sound processor, which can be worn on an adhesive gel pad or a designated softband. In children over five years of age, the Cochlear BAHA® SoundArc may be used in conjunction with the Cochlear BAHA® Sound processors and is worn on the posterior aspect of the head as an alternative to the headband.

Clinicians should encourage early use of these worn bone conduction devices for infants with CHL. In bilateral microtia/atresia cases, infants should be fit for a device by an audiologist by 4 months of age (8). In patients with *unilateral* microtia/atresia, where the non-microtic ear has normal hearing, a device consultation with a pediatric audiologist to discuss bone conduction options and encourage early use to facilitate early adaptation to hearing aid use. Although children under three years old are seldom exposed to challenging hearing environments, it is the experience of most centers that successful use of bone conduction aids in school-aged children correlates with earlier fitting and adaptation. Parents should be made aware that they may not notice a behavioral difference in the early fitting of bone conduction aids, and some may feel that it “doesn't make a difference.” The goal of the early fitting is early adaptation to amplified sound for sensory integration into the child's experience in preparation for school age. While there is an increasing body of literature that indicates that the lack of sound localization in children with unilateral hearing loss is associated with increased difficulties with speech recognition in noise often found in the typical classroom environment (12–17), whether or not children with unilateral hearing loss from aural atresia actually gain benefit from amplification is still an emerging area of research with at least one group finding that bone conduction devices improved both sound localization and speech recognition in children with unilateral aural atresia (18).

Parents should be made aware that there may be challenges with insurance coverage for bone conductive hearing devices in patients with unilateral microtia. They should also be counseled that toddler years can be challenging for successfully using worn devices, and a goal of 2–4 h a day use is encouraged until the child is more tolerant of more extended periods of use. Many programs have the minimal goal of daily use of the hearing device while in school.

### Surgically implanted hearing amplification options

**Figure 6:**
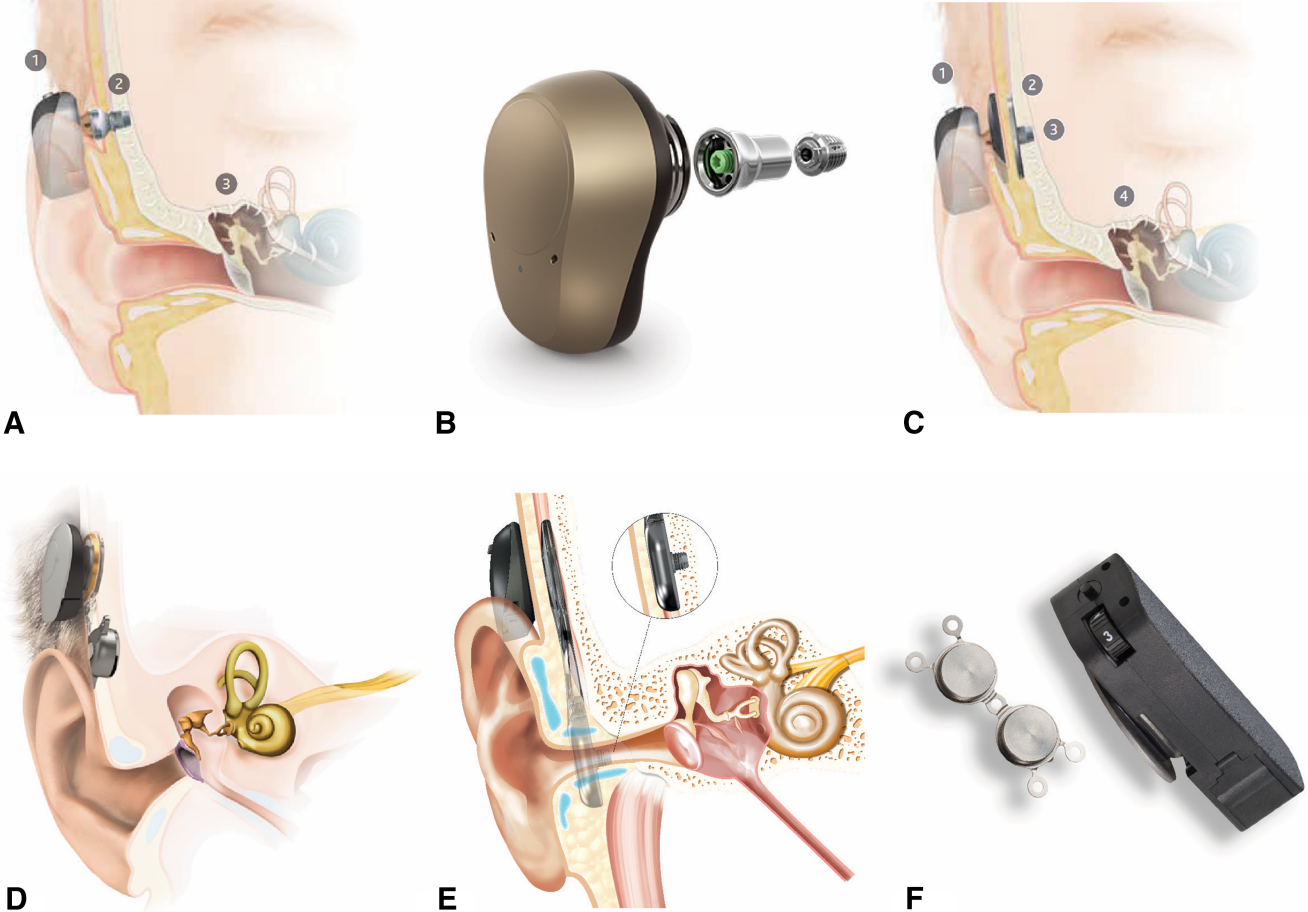
Surgical bone conduction devices. (**A**) Cochlear BAHA^®^ with osseointegrated percutaneous abutment. (**B**) Ponto^®^ with abutment and screw for osseointegration. (**C**) Cochlear BAHA ATTRACT^®^ with subcutaneous magnet. (**D**) Med-El BoneBridge^®^ with subcutaneous magnet. (**E**) Cochlear OSIA^®^ with subcutaneous magnet. (**F**) Medtronic magnetic implant.

Once patients reach five years of age, they are candidates to use BCSP with a surgically placed osseointegrated implant retention system, using either a subcutaneous magnet or a percutaneous abutment (Figure 6). This implant allows the child to use a bone conduction sound processor without a softband or an adhesive gel pad.

The abutment type (BAHA**®** Connect, Ponto) and the magnetic systems (BAHA**®** Attract, Medtronic Magnetic Implant) have a similar osseointegrated implant in the skull, but each allows the sound processor to connect to the implant differently. The abutments penetrate the skin, while the magnetic systems do not.

Overall, transcutaneous magnetic systems allow for eliminating an abutment that penetrates the skin. However, the hearing gain is less with a magnet. Different magnet strengths are available to accommodate for varying scalp thickness, though the device may fall off with weaker connections. Occasionally, the scalp needs to be surgically thinned to accommodate the magnetic connection.

In contrast, abutments allow direct bone conduction without any loss of vibration across the skin. Abutments also offer a more secure attachment of the processor to the implant, which can be beneficial for an active child on the playground. However, the skin-abutment interface is susceptible to local skin reactions such as recurrent infections and skin overgrowth. Rates of complications of abutments requiring revision surgery are as high as 44.4% percent, with implant loss ranging up to 25% in pediatric patients (19,20).

Active implants with direct bone conduction without skin penetration (Osia**®**, BoneBridge^TM^) are currently FDA approved for patients 12 years of age and older. At the time of this writing, successful implantation in younger patients has been reported both in and outside of the United States (21,22). These provide optimal hearing amplification without penetrating the skin. These devices also rely on transcutaneous magnetic systems to connect an external microphone. However, the bone conducting transducer is the component implanted under the skin. This allows for separation of the microphone and the active component of the hearing device, eliminating the commonly encountered issue of feedback without the loss of amplification across the scalp skin or issues with skin complications.

### Implanted hearing device candidacy

Close consultation with the audiology team and the family is essential to determine a patient's candidacy for a surgically implanted hearing device. A child with a history of poor compliance with a softband-retained hearing device, for instance, may not be a good candidate for an implanted device, particularly if the patient does not feel the benefit from the device, does not like the sound, or does not want to wear any device that might attract attention. A child who is a good candidate for ear canal reconstruction may not want to proceed with an implanted device.

### Timing of hearing implant surgery

The surgeon placing an osseointegrated implant for hearing management should collaborate with the reconstructive surgeon so that the implant surgery does not compromise the fascial flaps or skin that may be critical for microtia repair. It may be preferable to place the implant after microtia reconstruction, though coordinated approaches allow for combined ear reconstructive and hearing augmentation procedures.

**Figure 7:**
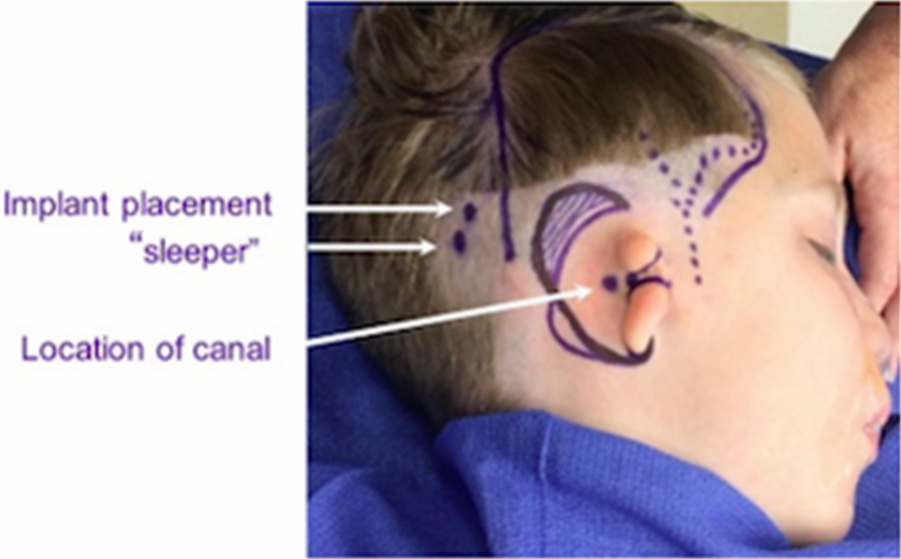
Surgical markings demonstrating course of the superficial temporal artery (dotted line), bone conducting hearing aid primary and “sleeper” implant sites (white arrows).

If placement is done before microtia reconstruction, it should be placed in the posterior temporal region. It is critical to preserve the vascular integrity of the superficial temporoparietal fascia during implant placement. Doppler of the superficial temporal artery at the time of surgery is recommended for safe placement of the implant posterior to the vessel's course (Figure 7). If a patient and family desiring microtia repair have not yet consulted with a microtia surgeon or have not yet decided on the timing and type of approach desired, deferring surgical implant of a hearing device may be prudent so that all surgeries can be coordinated.

If a non-surgical, adhesive retained prosthetic ear is desired, some osseointegrated hearing implants can be done at any time after five years of age. If a surgically retained prosthetic (i.e., Vistafix®) is planned, these surgeries can be done concurrently.

If a family is proceeding with alloplastic ear reconstruction, the osseointegrated hearing implant can be placed simultaneously with the alloplastic reconstructive surgery or three months after the ear has healed. For autologous cartilage repair, the implant can be placed concurrently during the stage of surgery in which the newly constructed ear is elevated, which allows access to the posterior auricular region through the same incisions. Alternatively, the osseointegrated hearing implant can be safely placed three months after the final stage of autologous cartilage repair.

### Hearing outcomes with hearing devices

Passive bone conduction devices worn on a softband (BAHA**®**, Ponto) can offer a similar pure-tone average as those worn with an implanted magnet (BAHA Attract®). However, implanted *active* bone conduction devices (Osia**®**, BoneBridge^TM^) provide better high-frequency gain (23–25). With improved high-frequency fidelity, patients can have improved speech discrimination scores with the active bone conduction implants compared to passive bone conduction devices.

Overall, hearing outcomes of bone conduction devices are reliable, come with less surgical risk, and are typically better than outcomes of ear canal reconstruction (26). However, they rely on a patient's compliance for use and have psychosocial impacts of wearing a hearing device.

### Aural atresia repair

Aural atresia repair requires creating an external auditory canal (EAC), a tympanic membrane, lining the neo-tympanum and EAC with squamous epithelium, and liberation or reconstruction of the ossicles of the middle ear. Although parents frequently hope that hearing can “be restored” with this surgery, it is more realistic to prepare for hearing improvement without complete closure of the air-bone gap over the long term (>5 years). Immediate hearing results can often be excellent but can degrade over time (26,27). It is reasonable for experienced surgeons to obtain improved air conduction thresholds with atresia repair for suitable candidates and create the anatomy that would allow the patient to accommodate a traditional behind-the-ear hearing aid.

Candidacy for aural atresia repair is based upon the ear-specific hearing assessment and CT findings. The most common scale used to describe the CT findings is the Jahrsdoerfer scale, a 10-point scale based upon the presence of 9 structures (28,29). Other anatomic factors that may be considered in determining a patient's candidacy for atresia repair include the position of the tegmen and other structures such as venous structures of the temporal bone and the middle ear volume (30–32).

It is absolutely critical that surgeons considering atresia repair closely communicate with the surgeon who plans to manage the microtia reconstruction for a coordinated surgical timeline that optimizes outcomes while minimizing complications.

### Optimal timing for aural atresia reconstruction impacted by the microtia reconstruction technique planned

*Ear Prosthetic:* atresia reconstruction surgery can be done at any time using an ear prosthetic, whether used with adhesive or surgically retained.

*Alloplastic Porous Polyethylene (PPE) reconstruction (e.g., Medpor®, SuPor®):* atresia reconstruction surgery is ideally done before *or* at the time of microtia reconstruction (concurrent surgery). Canal surgery *after* alloplastic porous polyethylene implant risks damage to the flap vascularity, exposure, and extrusion of the implant with or without infection, which is poorly tolerated by alloplastic implants (7, 33).

*Autologous costal cartilage reconstruction:* atresia reconstruction surgery is ideally done *after* microtia reconstruction with cartilage to maintain the viability of the overlying local skin and subcutaneous tissue, which covers the cartilage construct in a tissue envelope (7, 33). However, in cases of canal cholesteatoma, this may not be possible, requiring surgical management of the cholesteatoma and atresia repair prior to auricular reconstruction. In these instances, fibrosis and vascular compromise across old incisions poses an increased risk to skin viability with the subcutaneous pocket of the microtia repair. There can be more difficulty creating a pliable skin pocket around the reconstructed canal due to scarring and limitations to the skin pocket near the newly created tragus.

### Hearing results for ear canal reconstruction & controversies of ear canal reconstruction

Careful patient selection, surgeon-specific training and skills, and meticulous technique at every operative step are required for successful ear canal reconstruction surgery (33, 34). Aural atresia can be graded by severity, ranging from a Type A stenosis to Type C total bony atresia (Table 3) (7). The stenoses and partial atresias are at much higher risk for cholesteatoma due to the presence of skin inside the remnant canal (33). However, these allow for surgical approaches that do not require circumferential skin grafting of the reconstructed canal and thus may heal more reliably with better hearing outcomes (35–37).

**Table 3:** Weerda Classification of Congenital Aural Atresia.

Type	Description
A Stenosis	• Both fibrocartilaginous and bony parts EAC present but narrow • Hypoplastic temporal bone and tympanic membrane • Normal or small middle ear cleft • Normal or mildly deformed ossicles
B Partial atresia	• Some part of fibrocartilaginous or bony EAC present • Atretic plate, tympanic membrane missing or rudimentary • Small middle ear space • Fixed and malformed ossicles
C Total atresia	• Both fibrocartilaginous and bony parts EAC as well as TM absent • Severely contracted or absent middle ear space • Absent or severely malformed ossicles

### Imaging

Careful analysis of the CT scan is critical for determining candidacy for successful ear canal reconstruction. Ears scoring six or less on the Jahrsdoerfer grading scale had only a 45% chance of achieving a postoperative speech reception threshold of 30 decibels (dB) or lower, while ears scoring seven or higher had an 89% chance (28,29). Absence of an oval window uniformly predicts poor hearing results (38). Mesotympanic volume measurement greater than 42 mm^3^ and middle ear volume measurement greater than 305 mm^3^ have been demonstrated to predict much better hearing results (30,31). Other anatomic factors that could make ear canal reconstruction impossible or more difficult include a low-lying tegmen, a large malleus-incus complex positioned directly lateral to the stapes rather than the usual anterolateral position, facial nerve obstructing the oval window, and facial nerve turning anterolaterally and obstructing the lateral surgical approach to the attic (39). Poorer hearing outcomes due to need for prosthesis placement has been found with the “boomerang” malleus-incus complex (40).

### Hearing outcomes

Average hearing gain from atresiaplasty is about 24 dB in appropriately selected patients, compared to 38 dB gain from osseointegrated implant-retained devices (26). Atresiaplasty achieves less than 30 dB pure tone average (PTA) or air-bone gap (ABG) in about 70% of patients if the hearing is assessed less than six months after surgery. However, this deteriorates to about 50% of patients assessed greater than 12 months after surgery (26,27). Patients with canal stenosis receiving surgery obtain hearing results approximately 10 dB better than patients with total bony atresia (35). Ultimately, these patients with canal stenosis typically require surgery to prevent canal cholesteatoma. Atresiaplasty can be done to create a patent canal which would allow the patient to accommodate an ear level hearing aid.

### Considerations for canal reconstruction

There is concern for recurrent canal stenosis that occurs in up to one third of patients postoperatively (33). When the ear canal is lined with a skin graft, cerumen and debris typically do not migrate laterally, and therefore the patient requires ongoing and lifelong otologic care for aural hygiene. Parents need to be educated on the required maintenance and care of the reconstructed ear canal. Tolerance of ear cleaning under a microscope in the clinic requires a certain level of maturity in the patient and should be a factor when considering aural atresia repair. Swimming may lead to issues of recurrent ear drainage (7%–24%) after canal reconstruction for atresia (41).

Ear canal reconstruction may be necessary rather than optional when there is canal stenosis and concern for cholesteatoma. The presence of canal cholesteatoma may necessitate atresia repair prior to the normal sequence for optimal microtia reconstruction.

Ultimately, an experienced atresia surgeon must candidly discuss the predicted hearing gain expected from surgery compared to the available implantable bone conduction hearing devices, as well as the option of fitting an ear-level air-conduction hearing aid in the reconstructed ear canal as a good means to further improve hearing.

### Reconstructive options for microtia

#### Observation

In discussing the spectrum of reconstructive options, observation should be presented with associated benefits and drawbacks. A select group of patients are satisfied and confident with the microtic ear, elect to wear their hair long and cover the ear, and/or prefer not to undertake the potential surgical and anesthetic risks.

#### External auricular prostheses

**Figure 8:**
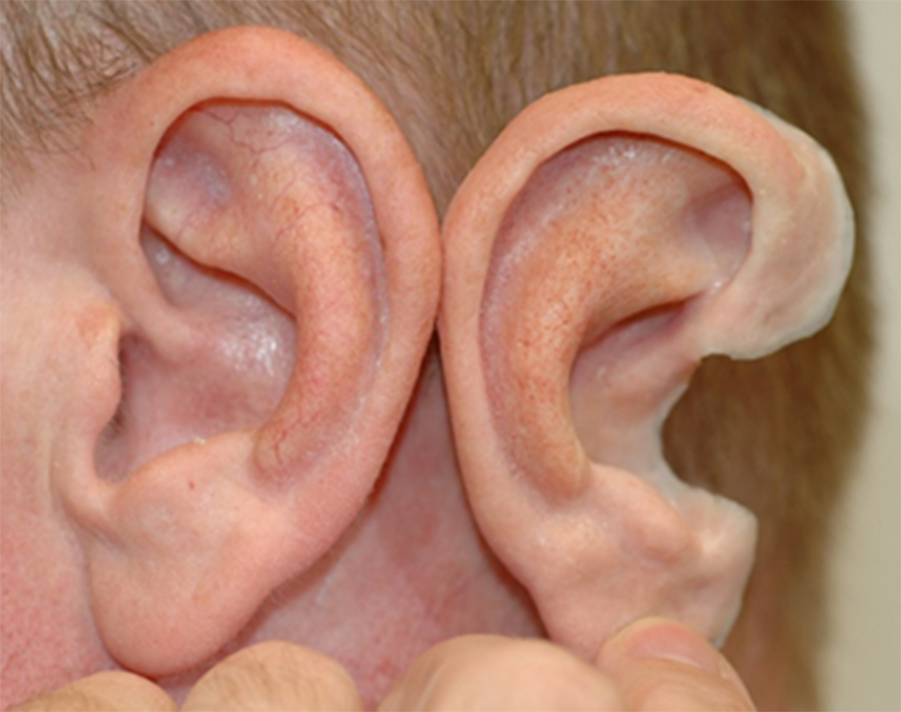
Silicone ear prosthesis compared to the contralateral well-developed ear.

A realistic silicone ear prosthesis can be made for individuals of any age (Figure 8). Parents desiring an ear prosthesis generally seek treatment between four and five years, or school-aged. The appropriate time to consider initiation of prosthetic use is the developmental age at which a child is able to care for and comply with wearing the prosthetic. Depending on the location of microtia ear, an ear prosthesis may be made to cover the ear and provide the desired symmetry with the contralateral ear using Computer-Aided Design and 3D printing. The anaplastologist factors in future tissue growth for the adhesive-held prosthetic to ensure maintaining a proper and secure fit. The anaplastologist then meticulously paints the prosthesis to match the skin (42). This expertise and skill is crucial for a satisfied patient. The prosthetic ear can also be made to cover an ear reconstruction that resulted in a poor cosmetic outcome. No surgery is required for an adhesive-held prosthesis, preserving the choice for more invasive interventions when the patient is mature enough to participate in the decision-making process.

Surgically placed osseointegrated implants can allow for a more secure attachment of the prosthetic by using a bar/clip, snap, or magnetic attachment, and can be implanted after the age of 5 years. The anaplastologist must be involved in the surgical planning as the location of the implant is paramount to a successful outcome of the prosthetic (43). Some patients may have chronic inflammation surrounding the percutaneous abutments, similar to those with osseointegrated percutaneous abutments for bone conduction sound processors. Placing the implants and/or excising the external ear will have serious ramifications, including precluding future repair, on microtia reconstruction. For this reason, it is imperative that the patient be mature enough to participate in the decision-making process.

A successful prosthesis should fit well and be secure enough to use the entire day without worry (44). A prosthesis can be pierced with an earring and enables the use of corrective glasses, oxygen tubes, hearing aids, and face masks. A well-made prosthesis should last two to three years and be remade with a new fitting to account for soft tissue changes. There may be some discoloration over time and degradation of the thin translucent edges of the prosthesis.

Other considerations include the potential need to remove the prosthesis during contact sports and swimming. Care of the prosthetic ear includes removal at night before sleep and daily cleaning.

Parents should be counseled that auricular prostheses and their replacement after normal wear and tear are generally covered by most insurances, though coverage varies. Ear prostheses provide the appearance of a normal outer ear with the psychological benefits of a symmetrical and realistic ear without the risk of surgery. The thought of a prosthesis inadvertently falling off may impact a child's self-image; this can be overcome with counseling and comparisons of the prosthetic ear to other commonly worn medical devices such as glasses and hearing aids.

#### Alloplastic porous polyethylene (PPE) ear reconstruction

**Figure 9:**
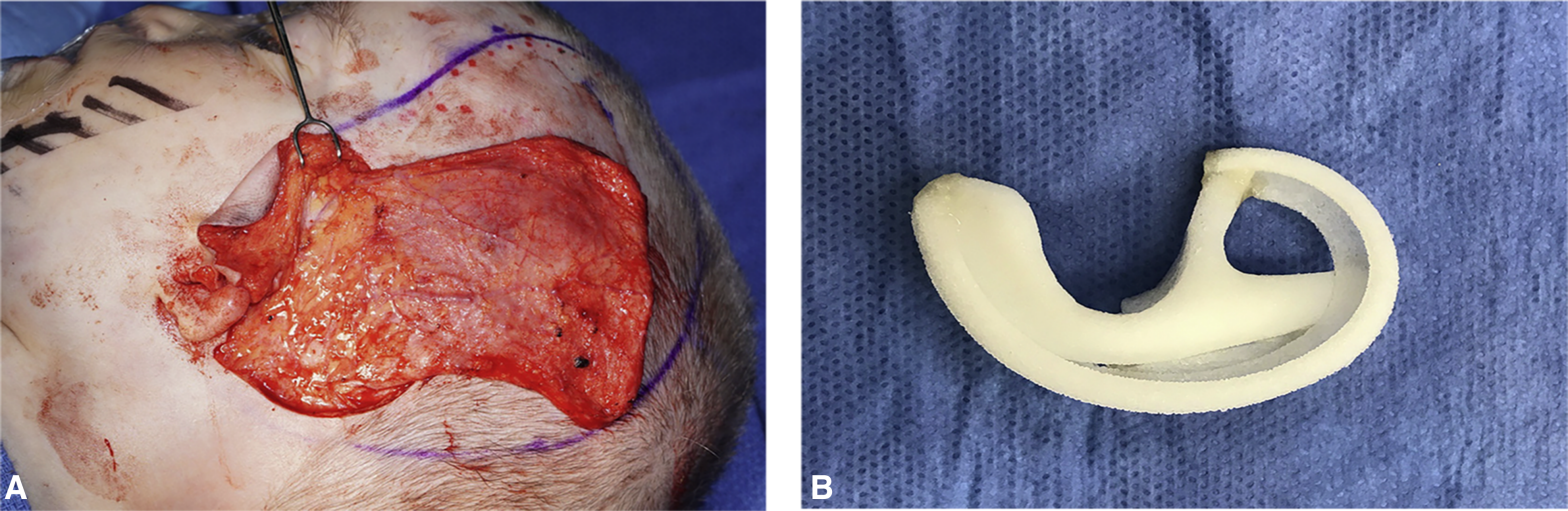
(**A**) Elevated temporoparietal fascia flap (TPFF). (**B**) Porous polyethylene auricular framework.

Alloplastic reconstruction, such as Medpor® and SuPor®, utilizes a high-density porous polyethylene implant covered with a well-vascularized fascial flap, typically the temporoparietal fascia flap (TPFF), which is then covered in remnant temporoparietal skin and skin graft (Figure 9). Alloplastic reconstruction is typically done as a single-stage surgery. Alloplastic reconstruction can be done as early as three years of age (45). Proponents of early reconstruction emphasize the avoidance of psychosocial bullying when the microtic ear is reconstructed prior to integration into school. Critics of early reconstruction emphasize the importance of the child's informed assent in a significant, life-altering, elective procedure. Quality of life improvements have been reported following alloplastic reconstruction as well (46).

The most compelling advantages of alloplastic reconstruction are that the reconstruction is done in one stage, and the age at the time of surgery is not dependent on the child's growth and size, as is with cartilage reconstruction (see below).

Disadvantages of alloplastic reconstruction include the overall risk of extrusion, fracture, and infection. Some feel this is amplified because the alloplastic reconstructed ear is insensate. Without sensation, the risk of injury, infection, and extrusion may be elevated and is lifelong. If extrusion does happen, the TPFF, often used for salvage in autogenous cartilage reconstruction, has already been exhausted in the initial repair. However, other fascial options such as the occipital artery flap may be utilized in salvage surgery. When the reconstructed ear's vascularity depends on a single vessel in a pedicled fascial flap, pressure may impact the viability. From the patient perspective, comfort with sleeping can be an issue that some children manage by sleeping with a special pillow around the ear to relieve the pressure.

An alloplastic ear reconstruction performed at a younger age will be larger than the opposite ear to account for future ear growth; patients and family must be advised of and agreeable to this initial size discrepancy for younger children. The first Medpor ear was done in 1991. Beyond that time span, we do not have more longitudinal outcomes or long-term data of this reconstruction technique (45).

Cases in which an alloplastic reconstruction may be most advantageous would be in instances of bilateral microtia, reducing the total number of surgeries and issues of symmetry, and for children who are extremely small for age (such as certain syndromic children), which otherwise would require the patient to wait for their teen years to have enough cartilage stock for adequate reconstruction. Also, for children with low hair lines, a technique that relies on coverage with TPFF is advantageous unless laser hair removal is pursued. Auricular reconstruction in older adults may be done with alloplastic reconstruction to eliminate the risk of having calcified cartilage stock which is difficult to carve.

#### Autologous cartilage reconstruction

**Figure 10:**
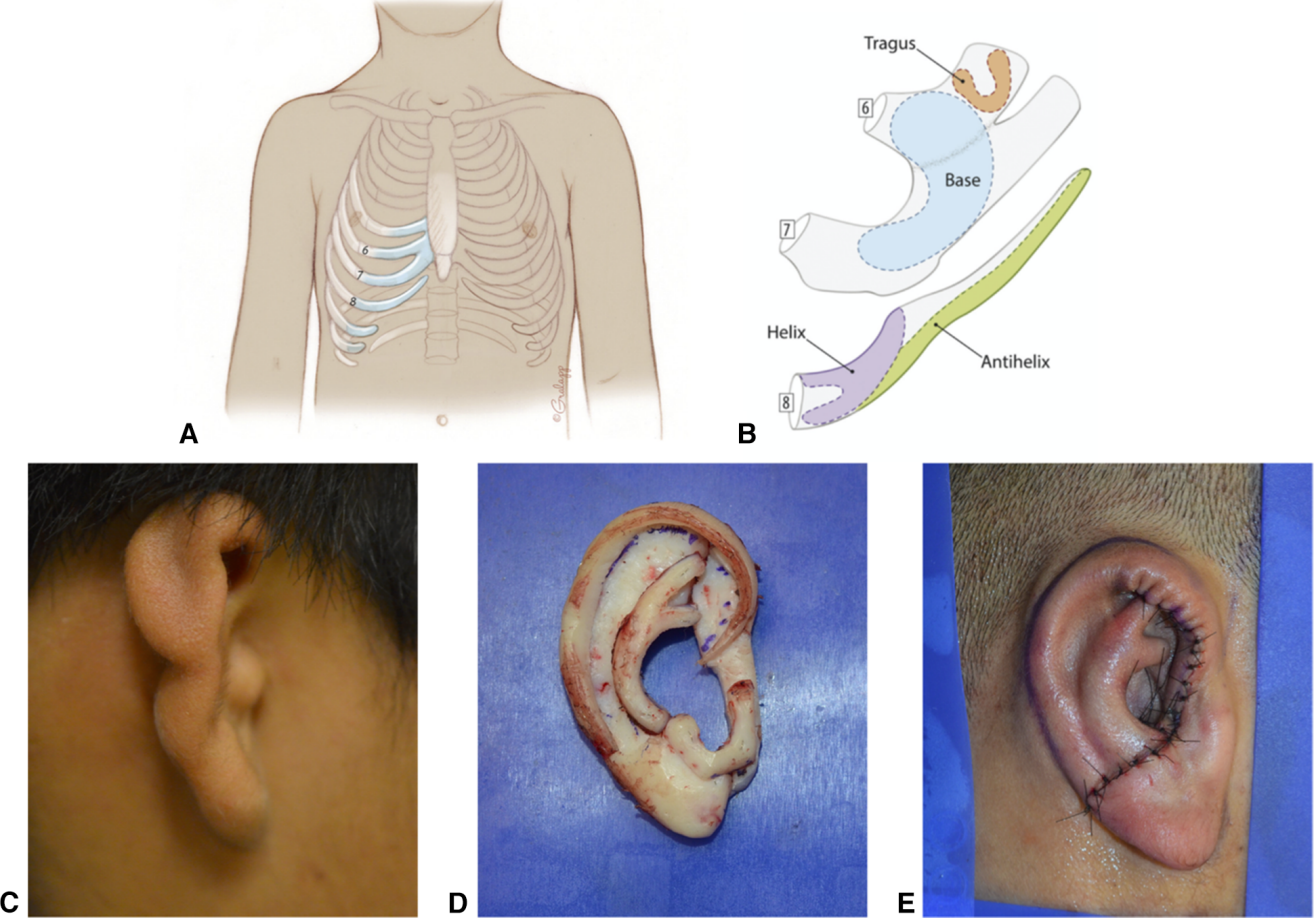
Autologous Cartilage Reconstruction. (**A**) Typical areas of costochondral cartilage harvest. (**B**) Example of auricular subunits created from costochondral cartilage. (**C**) Pre surgical picture of microtic ear. (**D**) Assembled costochondral auricular framework. (**E**) Immediate postoperative appearance after Stage 1 costochondral cartilage reconstruction.

Use of a patient's own, or “autologous,” rib for auricular reconstruction is regarded by many as the gold standard for surgical microtia reconstruction (Figure 10). Decades of follow-up have shown that a properly executed surgery can provide patients with a stable, life-long reconstruction. This surgery is completed in 2–4 stages, depending on the surgeon's technique. Typically, this is started when the patient is at least six years of age.

Tanzer introduced the surgical techniques, which Brent subsequently refined to achieve acceptable autologous ear reconstruction results (47). Variations on Brent's 4-stage technique remain the most utilized methods in the United States. With the Brent method, a smaller quantity of cartilage than other techniques allows for reconstruction beginning around six years of age. Nagata and Firmin described a 2-stage autologous approach that imparts greater detail but necessitates larger cartilage volumes. Reconstruction using Nagata and Firmin techniques is typically offered at later ages when the child had a chest circumference of at least 60 cm, the standard indicator of sufficient rib stock (48–52).

An advantage of cartilage reconstruction is the extensive knowledge base that has been amassed due to the broad use of this approach worldwide. The reconstructed ear has sensation and vascularity compared to an alloplastic ear. Cartilage reconstruction allows for the creation of an individualized ear, which is carved by the surgeon, to create symmetry with the opposite ear. This is ideal for various forms of microtia, particularly grades of microtia where part of the remnant ear can be saved, and part of the defect is reconstructed, such as in grade 2 microtia. Cartilage reconstruction allows the surgeon to reconstruct only the missing subunits of the ear, preserving part of the microtia ear as desired, which may not be possible with alloplastic techniques.

Some argue that an older child of age 6–10 years can be more involved in the decision-making process, more invested in the surgery, and a more active participant in the care of the ear. In addition, the older child tends to have the opposite ear very close to adult size, allowing for a true size match of the reconstructed ear rather than an approximation based on projected growth. Others argue that later reconstruction in school-aged children can lead to poor self-image and self-concept, though the age that this occurs is controversial and likely different for every child (53).

One disadvantage of cartilage techniques is the requirement for 2–4 stages, separated in time by 3–4 months, depending on the surgeon's technique. The earliest timing of surgery depends on the patient's chest size to allow for enough cartilage rib stock to create the ear, therefore necessitating a longer waiting period before surgery. There is also donor site morbidity at the time of surgery and a scar along the chest, though most patients do not report long-term adverse side effects for donor site issues. Patients do not have chest wall deformities or limitations in future activities or sports after rib cartilage harvest. Long term complications reported include framework resorption and wire extrusion, though overall, comparisons show less long-term complications using autologous cartilage compared to porous polyethylene (54). Pneumothorax is an uncommon short-term complication of rib harvest, and typically easily treated and resolved without lengthening hospital stay or causing any long term consequences.

#### Timing of cartilage reconstruction

Several factors should be considered in the timing of cartilage-based reconstruction. The patient's age, size, and maturity are all critical factors.

Age guidelines are general, as many factors such as genetics, diet, and medical comorbidities are strong determinants of a child's size. A chest circumference of 60 cm, measured at the xiphoid level has classically been the minimum indicator of the adequacy of rib stock. Classically, CT scans of the chest were performed to evaluate the costal cartilage, allowing for a view of the synchondrosis and evidence of calcification. However, with increasing awareness of radiation exposure in children with computerized tomography, this has been less practiced. More recently, ultrasound of the cartilage has been reported to accurately assess rib dimensions without the need for ionizing radiation exposure (55). Multidisciplinary evaluation and careful conversation with the child and family also help determine psychological and anatomic readiness.

Timing is critical when considering aural atresia repair in relation to auricular reconstruction. The desire of many families for aural atresia repair at a young age while deferring auricular reconstruction until a later time must be resisted if one wants to pursue autologous cartilage reconstruction. The local tissue changes resulting from atresia repair or a poorly placed osseointegrated implant can complicate or preclude successful cartilage reconstruction (56).

#### Surgical expertise

Any technique, alloplastic or cartilage reconstruction, requires a high level of surgical expertise with an experienced surgeon to achieve reliable outcomes. Surgeons who perform microtia reconstruction should regularly perform the surgery and have advanced training.

### Prominent techniques using autologous cartilage for microtia reconstruction

There are three primary techniques reviewed here, which are commonly used in variations customized by the reconstructive surgeon (Table 4). Historically, Tanzer described a six stage technique which serves as a foundation of the Brent technique (57).

**Table 4:** Stages of common autologous cartilage reconstructive techniques.

	Stage 1	Stage 2	Stage 3	Stage 4
**Brent Technique *traditional***	• Costal cartilage harvest • Creation of framework (Body of framework, helical rim, anti-helix) • Inset of framework into retroauricular skin of microtia remnant	• Lobule is transposed onto framework from microtia remnant	Elevation of ear and placement of posterior auricular skin graft	• Tragus is created from contralateral conchal cartilage free graft • Conchal bowl is deepened
**Brent Technique *modified***	• Costal cartilage harvest • Creation of framework *with* tragal strut • Inset of framework into retroauricular skin of microtia remnant	• Lobule is transposed onto framework from microtia remnant	Elevation of ear augmented with banked costal cartilage covered with fascial flap, and skin graft	N/A
**Nagata/Firmin**	• Costal cartilage harvest • Creation of full framework • Inset of framework into retroauricular skin • Lobule transposition, inset of framework into lobule	• Ear is elevated • Elevation augmented with banked costal cartilage, covered with a facial graft and skin graft	N/A	N/A

### Brent technique

This is the first modern microtia technique to gain wide popularity as adapted from Tanzer. It is traditionally accomplished in 4 stages (58).

• 1st stage: Costal cartilages 6–9 are harvested and carved. The cartilage framework is then placed deep to the retro and peri-auricular skin of the remnant.
• 2nd stage: The lobule of the microtia remnant is transposed to the framework.
• 3rd stage: The ear is elevated with the use of a skin graft placed in the posterior aspect of the ear and the advancement of the retroauricular skin.
• 4th stage: A tragus is created from contralateral conchal cartilage, and the conchal bowl of the construct is deepened.

More recent variations on the Brent technique have reduced the number of stages to 3 by combining the tragus formation with the first stage, creating a tragus with the framework, and often combining the final stage with the placement of an osseointegrated hearing implant (59).

### Nagata/firmin techniques

Satoro Nagata revolutionized auricular reconstruction with the description of a 2 staged technique, where reconstruction of the tragus and transposing of the lobule are both done in the first stage (48–50). Françoise Firmin went on to describe what many consider a modified-Nagata technique, characterized by projection pieces of the framework and a formal classification of skin approaches which she calls Type 1, 2, 3a and 3b. This technique provides a standardized approach to auricular reconstruction which takes into account the microtia remnant and missing contours to be reproduced (51,52). These techniques use autologous costal cartilage to reconstruct the ear in 2 stages (48–51, 60–61).

• 1st stage: Costal cartilage 6–9 are harvested and carved to create a framework that includes all the structures of the ear that need to be reconstructed, including the tragus. The cartilage framework is then placed deep to the retro and peri-auricular skin of the remnant with concurrent lobule transposition. Different grades of microtia require variations in skin approaches.
• 2nd stage: Elevation of the ear is performed by incising the skin around the framework. Additional cartilage is placed posteriorly to project the ear. This is either obtained through a second harvest or is derived from excess cartilage banked in the soft tissues of the chest at the time of the first operation. In the Nagata technique, the cartilage used for elevation in the postauricular sulcus is covered with vascularized TPFF and a skin graft.

Firmin describes four techniques to elevate the ear and recreate the retroauricular sulcus, which she categorizes as Type A-D elevation. Type A is the standard Nagata technique utilizing the TPFF fascia to provide vascularity to the elevation cartilage. Type B is the Brent technique, where no cartilage is used, the posterior ear is elevated and covered with a skin graft, and the retroauricular skin is advanced. Type C describes the use of random, posteriorly based fascia from the mastoid region, turned over to provide vascularity to the elevation cartilage, which is then covered by a skin graft. Type D is known as the tunnel technique, where tunnels are created within the enveloping soft tissue beneath the framework, where cartilage blocks are placed to prop the ear forward. As these are tunneled, they do not require coverage with a facial flap. The retroauricular sulcus is then covered with a skin graft. The elevation technique chosen depends on patient factors such as the degree of projection of the other ear, the hairline, and the amount of cartilage available for the second stage.

Ruhong Zhang et al. described the quality of retroauricular fascia for use in staged auricular reconstruction, finding that more superiorly based fascia has better vascularity, elasticity, and lymphatics (62). This led to his description of a posterior-superior fascial flap that is rotated down to cover the elevation cartilage (63).

The advantage of the 2 stage techniques, though they require more expansive cartilage frameworks, is a reconstructed ear with more detail and depth. However, these techniques are more demanding on the skin and may result in increased risk of wound complications such as small areas of dehiscence and problems with skin viability postoperatively. These can typically be managed with local wound care or closures. The advantage of the Brent technique is that each stage allows the skin to heal in between, thus reducing the risk of wound complications.

### What is a general timeline for the hearing and reconstructive needs of a child with microtia/atresia?

**Figure 11:**
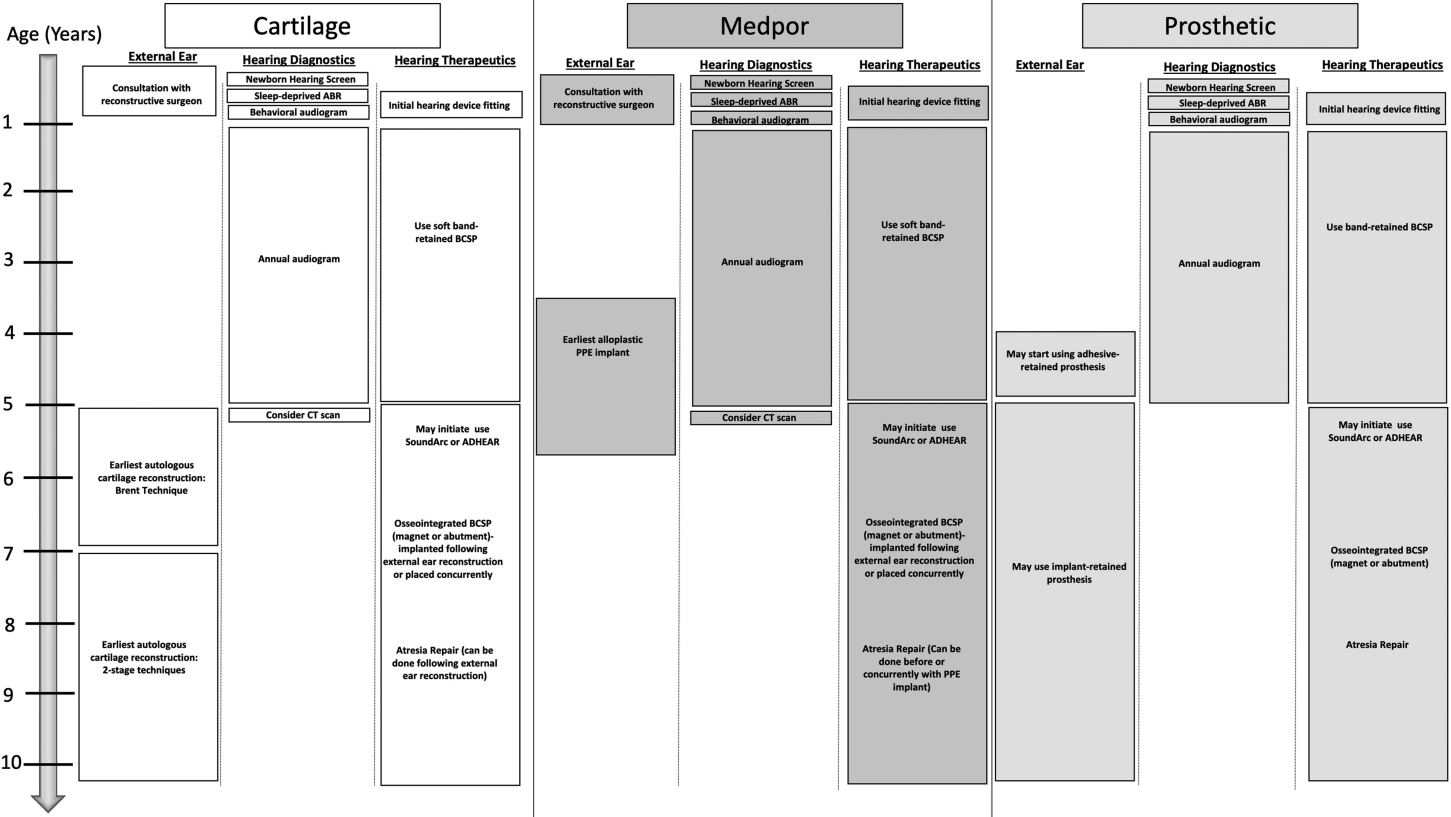
Recommended timing of key events for the hearing management and reconstruction of microtia and aural atresia.

Figure 11 describes the recommended timing of key events for the hearing management and reconstruction of microtia and aural atresia (Figure 11).

Age: Intervention recommended for the hearing needs of the child

• Newborn: Hearing screening
• 1–3 months: Sleep deprived ABR
• 6–9 months: Behavioral audiogram
• 3–6 months: Consultation on hearing devices (bilateral cases should be fit with softband device by 4–6 months of age)
• 12months-5 years: audiological testing and surveillance every 6–12 months
• 5 years: Consider CT scan for hearing implants and canal reconstruction candidacy. Consider waiting for surgery to implant bone conduction device until after or in coordination with concurrent microtia reconstruction. Consider Cochlear^TM^ BAHA**®** SoundArc or Adhesive gel modes of Bone conduction sound processor use.

Age: Intervention recommended for microtia reconstruction

• Newborn: Consultation with reconstructive surgeons for parental counseling regarding reconstructive options for the future
• 3–5 years: Earliest age for consideration for alloplastic porous polyethylene reconstruction. Any ear canal surgery should be done *before* or concurrently with alloplastic reconstruction
• 5 years: Earliest age for an osseointegrated anchor for prosthetic use. Prosthetics may be worn with adhesive prior to age 5.
• 5–9 years: Earliest age for autologous cartilage reconstruction. Any ear canal surgery is ideally done *after* microtia reconstruction.

## Conclusions

Caregivers of children born with microtia and atresia face numerous options for their hearing and reconstructive needs, the recommendations for which are often provided by separate and non-communicating disciplines. Plans for hearing augmentative devices, hearing reconstructive surgeries, and auricular reconstruction should be provided in a cohesive manner, with close communication between the atresia repair/hearing rehabilitation surgeon and the microtia reconstruction surgeon. This guideline aims to integrate the hearing and reconstructive recommendations with a timeline that allows for the understanding of all options available, realizing that every patient is unique and requires an individualized plan.
